# The Role of Fc Receptors on the Effectiveness of Therapeutic Monoclonal Antibodies

**DOI:** 10.3390/ijms22168947

**Published:** 2021-08-19

**Authors:** Patricia Gogesch, Simone Dudek, Ger van Zandbergen, Zoe Waibler, Martina Anzaghe

**Affiliations:** 1 Division of Immunology, Section 3/1 “Product Testing of Immunological Biomedicines”, Paul-Ehrlich-Institut, D-63225 Langen, Germany; Patricia.Gogesch@pei.de (P.G.); Zoe.Waibler@pei.de (Z.W.); 2 Division of Allergology, Section 5/3 “Clinical Allergology”, Paul-Ehrlich-Institut, D-63225 Langen, Germany; Simone.Dudek@pei.de; 3 Division of Immunology, Paul-Ehrlich-Institut, D-63225 Langen, Germany; Ger.vanZandbergen@pei.de; 4 Institute for Immunology, University Medical Center of the Johannes Gutenberg University of Mainz, D-55131 Mainz, Germany; 5 Research Center for Immunotherapy (FZI), University Medical Center, Johannes Gutenberg-University Mainz, D-55131 Mainz, Germany

**Keywords:** therapeutic monoclonal antibodies (mAbs), Fcγ receptor (FcγR), antibody dependent cellular cytotoxicity (ADCC), antibody dependent cellular phagocytosis (ADCP), effector function, modes of action (MoA)

## Abstract

Since the approval of the first monoclonal antibody (mAb) in 1986, a huge effort has been made to guarantee safety and efficacy of therapeutic mAbs. As of July 2021, 118 mAbs are approved for the European market for a broad range of clinical indications. In order to ensure clinical efficacy and safety aspects, (pre-)clinical experimental approaches evaluate the respective modes of action (MoA). In addition to antigen-specificity including binding affinity and -avidity, MoA comprise Fc-mediated effector functions such as antibody dependent cellular cytotoxicity (ADCC) and the closely related antibody dependent cellular phagocytosis (ADCP). For this reason, a variety of cell-based assays have been established investigating effector functions of therapeutic mAbs with different effector/target-cell combinations and several readouts including Fcγ receptor (FcγR)-mediated lysis, fluorescence, or luminescence. Optimized FcγR-mediated effector functions regarding clinical safety and efficacy are addressed with modification strategies such as point mutations, altered glycosylation patterns, combination of different Fc subclasses (cross isotypes), and Fc-truncation of the mAb. These strategies opened the field for a next generation of therapeutic mAbs. In conclusion, it is of major importance to consider FcγR-mediated effector functions for the efficacy of therapeutic mAbs.

## 1. Introduction

Monoclonal antibodies (mAbs) are immunoglobulins (Ig) which represent a therapeutic tool for malignancies (hematological or solid), transplant rejection, autoimmune diseases (e.g., rheumatoid arthritis (RA) or multiples sclerosis (MS)) and viral infections (with, e.g., Ebola, influenza, or respiratory syncytial virus (RSV)) [1,2,3,4]. Among the five isotypes of Ig which are IgM, IgG, IgA, IgD, and IgE, IgG is the most abundant isotype within the blood, mainly because of its extremely long serum half-life [5]. Antibodies consist of two heavy chains and two light chains, while each heavy chain associates with a light chain via disulfide bonds and non-covalent interactions to form a heterodimer. Both heterodimers in turn are paired through disulfide bonds between the heavy chains. Each heavy and light chain heterodimer includes the antigen binding fragment (Fab) which is composed of the variable and constant region of the light chain (VL and CL), the variable region of the heavy chain (VH), and the CH1 domain of the constant region of the heavy chain. VL and VH at the tip of the antibody are forming the antigen binding site. C-terminally located to the Fab is the hinge region and the crystallizable fragment (Fc), which includes the CH2 and CH3 domains [6,7]. All mAbs exert their function by direct targeting via their antigen-specific Fab arm. Additionally, Fc:Fc receptor (FcR) interaction can modulate their modes of action (MoA) [2,4,8]. The human FcRs belong to the Ig superfamily and are type I transmembrane glycoproteins, which bind to the Fc tail of antibodies. They comprise receptors for all five antibody classes: FcµR (IgM), FcγR (IgG), FcαR (IgA), FcδR (IgD), and FcεR (IgE) [2,8,9]. Hereby, FcγRs are of specific therapeutic relevance as all approved mAbs belong to the IgG class [5,7,8]. 

Humans possess two classes of FcγR, the activating and the inhibitory receptors. While activating FcγRs transmit their signals via the immunoreceptor tyrosine-based activation motif (ITAM), inhibitory FcγRs exert their function through immunoreceptor tyrosine-based inhibitory motifs (ITIM) [2,4,10,11]. The activating FcγRs are FcγRI (CD64), FcγRIIa (CD32a), FcγRIIc (CD32c), and FcγRIIIa (CD16a), while FcγRIIb (CD32b) is the only inhibitory FcγR and able to block cellular activation in order to downmodulate immune responses. FcγRIIIb (CD16b) is expressed on neutrophils and attached to the cell membrane via a glycophosphadylinositol (GPI) anchor. Despite lacking an intracellular signaling domain and ITAMs, it is capable of promoting neutrophil activation and killing of target cells. Accordingly, FcγRIIIb is often considered as activating FcγR [2,4,8]. In addition, Brambell et al. discovered an intracellular trafficking receptor for IgG in 1966. This receptor was called the neonatal FcR (FcRn) and represents a non-covalent heterodimer of major histocompatibility complex (MHC) class I like heavy chain and a β2 microglobulin light chain. Due to its ability to bind to the Fc domain of IgG at acidic pH in the endosome, it is capable of protecting IgG from degradation and prolongs their serum half-life [5,12,13].

FcγRs can be classified as low affinity receptors, that can bind IgG when multimeric (presented in immune complexes, aggregated, or opsonized), or high affinity receptors, which can bind monomeric Ig [10]. FcγRI is the only receptor binding monomeric IgG with a high affinity while all other FcγRs bind monomeric IgG with low or intermediate affinity. Thus, the formation of immune complexes allows high avidity Fc binding of multimerized IgG to low or intermediate affinity FcγRs. These are in turn crosslinked, leading to cell activation [4]. FcγRs are mainly expressed on cells of the innate immune system, such as antigen presenting cells (APC) or natural killer (NK) cells, but can also be found on activated T cells, endothelial cells, microglial cells, osteoclasts, and mesangial cells [8,14,15,16]. An overview of the expression profile of the different FcγRs is given in Table 1.

**Figure 1:**
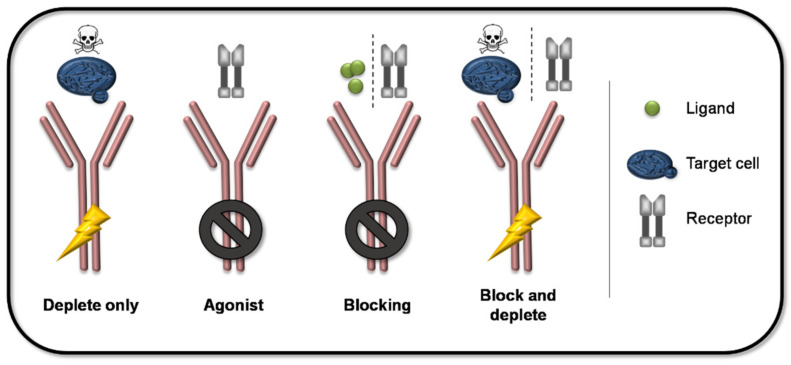
Principle modes of action of mAb. The combination of the antigen-specificity and different Fc-mediated effects result in different MoA of mAbs. Depleting mAbs induce the elimination of the target cell via FcγR-mediated ADCC or ADCP. Agonists bind with higher affinity to a respective receptor than its ligand without eliciting FcγR-mediated effects. Blocking antibodies bind ligands or receptors without engaging FcγR activation and thereby inhibit the respective downstream events. Furthermore, mAbs can be designed to specifically block receptors and induce depletion of target cells at the same time.

FcγRs can exert their function either via direct effects such as the induction of antibody dependent cellular cytotoxicity (ADCC) and the closely related antibody dependent cellular phagocytosis (ADCP), or via indirect effects such as induced cytokine production, enhanced antigen presentation, and the induction of reactive oxygen species (ROS) production (see graphical abstract) [2,17]. Accordingly, besides Fab-mediated antigen-specific targeting, different optimization strategies of the interaction between the Fc part of a mAb and an FcγR give rise to a variety of MoA. An overview of the principle MoA of mAbs are given in Figure 1. Combinations of Fab-mediated antigen-specificity and the activation/inhibition of FcγR-mediated effects result in different MoA such as depletion, agonizing, blocking, as well as the combination of blocking and depletion. This variety enables the development of various treatment options for a wide range of clinical indications. In addition, a significant effort has been made to improve therapeutic efficacy and safety via Fc-engineering in order to modify the desired Fc-mediated effector functions of therapeutic mAbs.

## 2. Clinical Development and Approval of Therapeutic mAbs

The first mAb approved for the European market was Orthoclone OKT3 (muromonab) in 1986 [18,19]. OKT3 is a murine mAb of the IgG2a isotype targeting CD3 which is expressed on all mature T cells and medullary thymocytes. It was extensively used in organ transplantation for both the prevention and treatment of rejection [20]. Since it has been withdrawn from the market due to severe side effects (see Section 3), MabThera, the α-CD20 antibody rituxumab, applied for the treatment of hematological malignancies such as Non-Hodgkin’s Lymphoma and Chronic Lymphatic Leukemia (CLL), is now the “oldest” therapeutic mAb in current use which remained unchanged since licensed in 1998. In the late 90s and during the early years of 2000, approximately 1–4 antibodies were approved per year with increasing approvals over the time and a peak in the years of 2017 and 2018 with 17 and 21 approvals, respectively. Since 2015, we have had an immense growth in the development and approval of new therapeutic antibodies covering multiple new antibody formats and new indications. As of July 2021, 118 therapeutic mAbs are approved for the European market [21].

### 2.1. Characterization of Therapeutic mAbs

As very complex and versatile molecules, therapeutic mAbs exert their effector function through different MoA: (i) specific binding of a soluble antigen (e.g., α- tumor necrosis factor [TNF]-α), (ii) specific binding of a target cell-bound antigen (e.g., α-CD20), (iii) specific agonizing or antagonizing with receptors in order to modulate immune responses (α- cytotoxic T lymphocyte-associated protein 4 [CTLA-4]). The MoA of mAbs are defined by different factors such as their binding activity and affinity to their target, the biological function of their target, and their effector functions which in turn affect their pharmacology and bio-distribution profile [22,23]. As a prerequisite for their clinical development, therapeutic mAbs are characterized regarding their functionality, their molecular characteristics, and their effector functions (as reviewed in [23]). 

The functionality of mAbs is defined by the interaction with their specific antigen, comprising parameters like the equilibrium dissociation constant and ligand binding per se. The latter can be analyzed by different methods such as Biacore, enzyme-linked immunosorbent assay (ELISA)-based analyses, and cell-based ligand-binding assays, which are important to assess biological post-binding downstream effects. 

For the molecular characterization of mAbs, primary, secondary, and higher order structures are determined by e.g., different mass-spectroscopy (MS) methods such as electrospray ionization (ESI)-MS, liquid chromatography (LC)-MS (peptide mapping), multi-angle laser light scattering (MALS), size exclusion chromatography (SEC) [24]), and fluorescence spectroscopy. Furthermore, mAbs are decorated with glycan structures at their Fc domains, which can be determined by high performance liquid chromatography (HPLC) or LS-MS (peptide mapping) analyses. Characterization of mAb-glycosylation patterns is of great importance, as they are known to critically affect the effector functions of the antibody, which are in turn directly associated with the pharmacokinetics (PK) and pharmacodynamics (PD) profile and thus, the clinical activity of the mAb [25,26,27]. Consequently, glyco-engineering of mAbs is up to now commonly performed in order to optimize their MoA or improve safety profiles (see Section 4). At the same time, the glycosylation patterns are one of the main sources for mAb heterogeneity [27,28,29]. Accordingly, non-clinical analyses assessing the contribution of afucosylation and galactosylation to the clinical activity of ADCC-inducing therapeutic mAbs revealed that within 54 batches of one mAb, just 26 could be clustered with similar afucose/mannose values [30]. To address this issue, pre-clinical analyses of glycan-mediated changes in mAb effector functions were collected to evaluate the glycan structure of mAbs in the context of critical quality attributes (CQAs) (reviewed by [27]). CQAs are defined as physical, chemical, biological, or microbiological properties or characteristics that must fulfill certain criteria. The obtained results must achieve an appropriate limit, range, or distribution to ensure the desired product quality, safety, efficacy, and PK/PD [31]. Hence, CQA are a component of quality-by-design approaches during product development, which aim to ensure manufacturing consistencies by raw and in-process material quality testing. The review concludes that the close relationship of mAb-glycosylation patterns with their clinical activity demonstrates the feasibility/necessity of their in depth characterization as a CQA [27].

### 2.2. Fc-Mediated Effector Functions as mAb Characteristic

**Figure 2:**
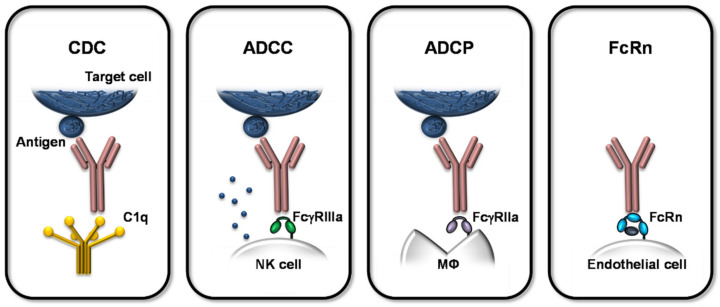
Overview of Fc-mediated effects of therapeutic mAbs. The Fc-mediated effector functions are classified into CDC, ADCC, ADCP, and binding to FcRn. During CDC, the Fc domain of the mAb interacts with complement component C1q, inducing the activation of the complement cascade and subsequent cytotoxic effects. ADCC is mediated via NK cells activated by the interaction of cell-bound FcγRIIIa with the mAb’s Fc part. The activation mediates cytotoxic effector functions resulting in death of the target cell. ADCP is induced by interaction of the mAb’s Fc part and FcγRIIa on macrophages (MΦ), which in turn augments their phagocytic activity. CDC, ADCC, and ADCP result in the depletion of the specifically bound target cell, whereas only ADCC and ADCP involve Fc:FcγR binding. FcRn is expressed on, e.g., endothelial cells and binds antibodies pH-dependently. This interaction is critical for the turnover and serum half-life of mAbs and it also mediates the transfer of maternal antibodies to the fetus across the placental barrier during pregnancy.

The Fc-mediated effector functions are classified into complement-dependent cytotoxicity (CDC), ADCC, ADCP, and interactions with the FcRn (see Figure 2). CDC is defined by the Fc interaction with complement component C1q, followed by the activation of the complement cascade leading to the release and activation of anaphylotoxins, opsonins, and other complement components, which can induce downstream immune responses by binding to their receptors on different immune cells [22,25,26,27]. 

ADCC and ADCP are two mechanisms mediated by direct interaction of the antibody’s Fc domain with the corresponding FcγR. ADCC is mainly attributed to NK cells, but also neutrophils and eosinophils, which exert their cytotoxic activities upon activation by the activating FcγRIIIa. ADCP is mediated by FcγIIa-activated macrophages leading to augmented phagocytosis [22,25,32]. These Fc:FcγR-mediated mechanisms define the MoA of therapeutic mAbs as the antigen-specificity of the mAb is combined with its effector function for selected clinical applications. In that course, target cells such as malignant/tumor or infected cells, bearing a specific surface antigen, are bound by the mAb with its antigen-specific Fab fragment and simultaneously crosslinked to the respective effector cell, expressing the matching FcγR. This bridging induces the FcγR-mediated activation of the effector cell, eventually leading to the elimination of the target cell by lysis (ADCC; e.g., obinutuzumab) or phagocytosis (ADCP; e.g., ofatumumab). 

For other MoA, the inhibition of Fc-mediated effector functions is desired. In these cases, mAbs are used to bind and thereby block, e.g., pro-inflammatory cytokines (e.g., TNF-α for the treatment of RA), disease-dependent pathological mediators (e.g., vascular endothelial growth factor (VEGF) in pathological angiogenesis), immuno-oncology (e.g., CTLA-4 inhibition to prevent downregulation of immune responses against cancer cells), or autoimmunity-related molecules (e.g., B lymphocyte stimulator (BLyS) for the treatment of patients with systemic lupus erythematosus (SLE)). In these inhibitory scenarios, concomitant induction of ADCC/ADCP and resulting exaggerated immune responses are adverse effects to be avoided. Accordingly, therapeutic mAbs are designed depending on the clinical context and the desired effector function/inhibition.

The effector functions of therapeutic mAbs are in close relationship with their PK/PD and clinical activity, hence their potency but also their safety since exaggerated effector functions or their unwanted inhibition can be translated into (severe) side effects (see Section 3). Furthermore, the PK/PD attributes of mAbs are also influenced by their ability to interact with FcRn. 

The FcRn is expressed on innate immune cells such as macrophages but also on endothelial cells and trophoblasts of the placenta [33]. It regulates antibody serum half-life and transfer of maternal antibodies via the placenta. Fc:FcRn interaction is influenced by the pH milieu. In the acidified endosome, mAbs bind to the FcRn and are thereby prevented from degradation. Instead, the FcRn-bound IgG is recycled to the cell membrane and released upon physiological pH. The high affinity of IgG to FcRn contributes to their long half-life [33,34,35]. Of note, this is apparently regulated independently from glycosylation patterns [36].

### 2.3. Safety Assessment

Besides their intrinsic heterogeneities due to their complex structure, therapeutic mAbs can attain extrinsic variations during their handling, e.g., in the course of their production and storage. All these potential variations emphasize the need to control each production step and the resulting characteristics of mAbs to provide batch-to-batch consistency, quality control, and reliable safety profiles [23]. 

The safety of therapeutic mAbs can be assessed on different levels. The requirements, which have to be fulfilled for the non-clinical safety assessment of mAbs, are defined in guidelines published by the International Council for Harmonization (ICH) or regulatory authorities such as the European Medicines Agency (EMA) and the Food and Drug Administration (FDA) [25,26,31]. In vitro studies can be used to assess immunopharmacology, potential of induction of cytokine release, potential for (dendritic) cell (DC) activation, and predictive immunogenicity testing (the production of anti-drug antibodies, ADA). In vivo, general toxicity studies, reproductive/developmental toxicity studies, as well as host resistance assays allow good laboratory practice (GLP)-compliant immunotoxicity assessment. Here, species selection and qualification is an important prerequisite for safety assessment. Surrogate mAbs reflecting the clinical activity or transgenic models have often been used for in vivo analyses in this context [37,38]. Murine IgG2a for example is known to have a high functional similarity to human IgG1 and is therefore capable of reflecting PK and Fc-mediated effector function [39,40]. Furthermore, Lo et al. developed a mutation-engineered mAb with a specific LALA-PG mutation (L234A, L235A, P329G). This mutation resulted in elimination of complement binding and fixation as well as silenced ADCC for both murine IgG2a and human IgG1. Hence, this variant harmonizes cross-species effector functions. Such modifications can contribute to a better translatability of clinical effects from pre-clinical model systems into the human context [39].

Besides studies with mice, non-human primates (NHP) are also used as model for in vivo safety assessment and are mostly treated with the mAb intended to be used in humans (no surrogate). However, differences in human and primate immune systems need to be taken into account. For example, it has been shown that NHP (macaques) do not express the T cell costimulatory molecule CD28 on effector memory T cells, which were identified to be critical key players inducing the cytokine storm after i.v. administration of the superagonistic mAb TGN1412 (closely described in Section 3 in this review). Beside other factors, these differences likely contributed to the massive side effects induced by TGN1412 as applied in the first in-human clinical trial in March 2006 [41]. Consequently, proper safety assessment requires representativeness of the in vivo models used and needs to be evaluated case by case depending on the mAb and its MoA. 

Bugelski and Martin investigated the concordance of preclinical and clinical pharmacology/toxicology of 15 selected approved mAbs across the human, murine, and NHP-systems based on peer reviewed literature, EMA “Scientific Discussions”, the FDA “Pharmacology/Toxicology Reviews”, and the respective prescribing information of the product. They could confirm good concordance of human PD with models of mice treated with surrogate mAbs or NHPs receiving the human mAb. However, poor concordance for human PD was concluded in genetically modified mice as well as for the assessment of adverse events in all three test systems [38]. 

Given the fact that therapeutic mAbs are rather “young” but promising pharmaceuticals on a growing market but possess inherent heterogeneities, it is not surprising that the establishment of standardized tests systems was pursued with great effort. Nevertheless, development, optimization, and harmonization of valid assays defining mAb-induced effector functions are still ongoing. As an example for an international standardization (IS) approach, Prior et al. developed a lyophilized candidate rituximab standard, which was analyzed regarding effector functions by 16 collaborating laboratories performing potency assays in compliance to the World Health Organization (WHO) international standard for mAb biological activity (11 ADCC, 5 cell-based binding assay, 1 direct apoptosis assay). This study reveals such a novel IS approach to be applicable promoting bioassay performance and data harmonization throughout different laboratories. This is not only useful for the assessment of mAb potency per se but also with regard to the fact that also the development of biosimilars is accelerating with expiry of more patents [42,43]. 

The EMA together with the European Directorate for the Quality of Medicines and Healthcare (EDQM) and the Official Medicines Control Laboratories (OMCL) Network initiated a program in 1997 with the aim to supervise the Centrally Authorized Products (CAPs) placed on the EU/EEA market. This program has been in operation since 1999 testing medical products upon their compliance with their authorized specification regarding all sections of the distribution chain. In that course, OMCLs verify if the used methods to analyze and characterize the respective medical product are appropriate and suitable. This is conducted in the frame of annual surveillance plans investigating approximately 30–40 products per year. The CAP Surveillance Project is the only coordinated market surveillance program in the EU/EEA including biological products for human and veterinary use and also includes regular testing of Active Pharmaceutical Ingredients [44,45]. CAP-testing includes a variety of potential test systems regarding the molecular and functional characteristics of mAbs. Additionally, a variety of potency assays are performed to determine the MoA including the effector function as well as the safety of the respective mAbs. CAP-tests include such bioassays in a standardized and validated manner to evaluate the potency or toxicity of the therapeutic mAbs.

### 2.4. Methods for Analyzing Fc:FcγR Interactions

**Figure 3:**
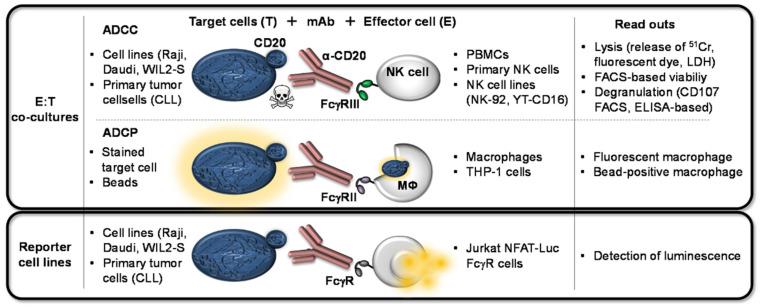
Cell-based assays used to assess the FcγR-mediated effector functions can be classified into co-culture systems involving effector plus target cells (E:T co-cultures) and assays including reporter cell lines. For the analyses of α-CD20-induced ADCC, several cell lines or primary tumor cells (such as Chronic Lymphatic Leukemia (CLL) cells) expressing CD20 can serve as target cells. Effector cell cultures for such assays can comprise crude PBMC cell cultures, primary NK cells, or NK cell lines. NK cell-mediated killing upon α-CD20:FcγRIIIa crosslinking can be detected via released labeling components in the cell culture supernatant (pre-labeled ^51^Cr, fluorescent dyes, or release of internal enzymes such as lactate dehydrogenase (LDH)). Further ADCC read outs are the detection of viability via FACS-based analyses or determining degranulation (CD107 by FACS analyses or ELISA). For the analyses of α-CD20-induced ADCP also several cell lines or primary tumor cells are used as target cells. FcγRIIa-expressing macrophages or macrophage-like cell lines (e.g., THP-1) are used as effector cells, which increase phagocytosis upon FcγRIIa-crosslinking with the mAb. When the target cells are pre-labeled with a dye or beads, their depletion can be traced by detecting fluorescent or bead-positive macrophages. Within reporter cell-based assays, the CD20-expressing target cells are co-cultured with Jurkat NFAT-Luc cells and the α-CD20 mAbs. When using this reporter cell lines, the FcγR-mediated effect upon crosslinking is replaced by the secretion of luciferase and can hence be determined by the intensity of luminescence.

The different effector functions of mAbs and their binding behavior to their respective FcγRs are pre-clinically assessed by a variety of assays reaching from ELISA-based binding assays to more complex cell-based potency assays including effector–target cell co-cultures. An overview of generally applicable cell-based assays in use to assess the FcγR-mediated effector function is given in Figure 3 using the α-CD20 IgG1 mAb (rituximab) as an example.

#### 2.4.1. ADCC

The commonly used methods to analyze ADCC and ADCP are based on co-cultures of effector cells and target cells. These target cells vary depending on the antigen-specificity of the therapeutic antibody. As α-CD20 mAbs belong to the “oldest” on the market, there are well established cell lines, which express CD20. For example, WIL2-S cells, a human B-lymphoblastic cell line (American Type Culture Collection (ATCC), CRL-8885), and Raji cells or Daudi cells both derived from a Burkitt’s lymphoma (Raji: ATCC, CCL-86; Daudi: ATCC, CCL-213) are applied in the assessment of Fc-mediated effector function. In addition, primary cells obtained from CLL patients can be used as target cells for CD20-specific mAbs [43,46,47,48,49,50,51]. 

For HIV-based ADCC/ADCP assays, CEM cell lines, derived from a T acute lymphoblastic leukemia (Expasy: CVCL_X622), are often used as they exert high capacities to bind HIV gp120 antigen [52,53]. A431 cells are deviated from squamous carcinoma (ATCC: CRL: 1555) and express the epidermal growth factor receptor (EGFR). Thus, A431 cells are applied in assays analyzing Fc-mediated effector functions of therapeutic EGFR-specific mAbs [54]. 

It is known that ADCC is mainly driven by NK cells. Hence, primary NK cells are often used as effector cells within ADCC assays, either alone or as part of a crude peripheral blood mononuclear cell (PBMC) culture isolated from human (or NHP) blood [47,50,52,53,54]. Although primary cells are considered closest to the physiological context, assays based on them are prone to donor-to-donor variations and also day-to-day variabilities. Mata et al. resumed work on a finding from 1984 [55], where cryopreserved cells thawed for use in in vitro assays were observed to perform better if they were allowed to rest (in the following defined as rested PBMCs) for one day at standard cell culture conditions (37 °C, 5% CO_2_) after thawing. In their ADCC assays using CEM.NKr as target cells and HIVIgs (pooled plasma from HIV^+^ individuals), Mata et al. revealed that rested PBMCs showed similar ADCC and NK cell activity when compared to freshly isolated PBMCs. In line with the finding from 1984, directly used PBMCs after freezing and thawing demonstrated decreased ADCC and NK cell activity when compared to the freshly isolated PBMCs even though expression of FcγRIII was not altered on NK cells by the freezing and thawing process [53]. The usage of cell-aliquots from one blood donor could facilitate minimization of variations. Another approach to circumvent donor-dependent variations, is the establishment of NK cell lines as effector cells such as NK-92 variants and YT-CD16 cells [30,46,48,51].

The readout of the ADCC assays is mostly defined by the specific lysis of the target cells, using respective controls of full lysis (e.g., Triton-X 100 treatment), unspecific lysis of co-cultures (without addition of the therapeutic mAb), and taking spontaneous lyses of untreated cells into account [30,46,48]. Determination of lysis was measured via ^51^Cr-release by target cells for many years [53,56]. Nowadays, such analyses can be based on the decrease of fluorescently labeled target cells (e.g., Cell-Trace Violet), increase of relative fluorescence units (RFU) in cell culture supernatants (e.g., Calcein-AM [46,52]), by measuring lactate dehydrogenase (LDH)-release [47,48,54], or using specific labeling systems [30,48].

Viability of target cells can also be detected by FACS-analyses of Annexin-V in combination with a viability stain. VanDerMeid et al. defined CLL target-cells as viable, if they were negative for both Annexin V and a dye that only penetrates dying cells [50]. Mata et al. also replaced the ^51^Cr release assay by a FACS-based assay to assess ADCC. The detection of CD107 (lysosomal-associated membrane glycoprotein-1, LAMP-1) as a marker for degranulation, perforin tracking, and granule movement [57] was used/established as surrogate marker for activation of NK effector cells [53]. Accordingly, CD107-detection was performed in another study in the scope of a protein-plate-bound degranulation assay where therapeutic mAbs/sera targets and NK effector cells were added [51]. Alternatively to these co-culture assays with PBMCs, primary NK cells, or NK cell lines, and reporter cell lines have been established. Upon co-cultivation of a Jurkat-16 cell line, stably transfected with nuclear factor of activated T cells (NFAT)-Luciferase and FcγRIIIa with target cells and respective mAb, Fc-mediated activation representative for ADCC can be read out as luminescence in the supernatant of these cultures [46,47,54]. Such reporter assays are also available for the murine system using Raji reporter cells and a corresponding bioassay kit [51]. Another assay combines a co-culture of Jurak/FcγR cells as effector cells labeled with calcein-violet and Daudi target cells labeled with calcein together with CD20-specific mAbs. Here, not the Fc-mediated effector function, but the bridging of Fc:FcγR can be tracked by the detection of calcein/calcein-violet double positive cells via FACS analyses. 

Miller et al. also established an ELISA-based bridging assay, which can be used as surrogate for ADCC-determination by FcγRIII-binding α-CD20-molecules [58]. Here, soluble target antigens are coated in a 96-well format and after incubation with the therapeutic mAb, glutathione-s transferase (GST)-tagged FcγRIIIa is added which in turn can be detected by a horseradish peroxidase (HRP)-conjugated α-GST antibody. Optical density hence reflects the occurrence of Fc:FcR-bridging representing the potential of the tested mAb to induce ADCC.

#### 2.4.2. ADCP

To assess the ability of a mAb to induce Fc-mediated phagocytosis, assays using the THP-1 cell line can be performed. Here, mAbs of interest are incubated with antigen-coated beads and THP-1 cells, a monocyte-like cell line, known to express FcγRI and RII [58]. Analyses of bead-positive THP-1 cells via FACS can be processed to reflect the phagocytic score [52,59]. Fox et al. used a similar approach/principle using murine bone marrow cells or bone marrow-derived monocytes which were incubated with Alexa-Fluor488-coated beads coated with Chikungunya virus (CHIKV) antigen p62. In their study, they could show in a mouse model for CHIKV-induced musculoskeletal disease that α-CHIKV therapeutic mAbs (a murine or a human variant) ameliorate the development of the disease. This abrogation of disease emerges as decreased foot swelling and is associated with reduced viral RNA in the affected muscle tissue and infiltration of immune cells including monocytes. The therapeutic effect was not observed in FcγR^-/-^ mice suggesting it is mediated by FcγR-activated phagocytic macrophages [60].

Additionally, for the analyses of ADCP, reporter gene assays are available with various target cell lines and are hence usable for different antigen specificities. Analogous to the ADCC reporter assays, crosslinked target cell lines secrete luciferase measurable as bioluminescence in the supernatant of the cell culture [32]. 

Other approaches use fluorescently labeled target cells for the incubation of effector cells and the respective therapeutic mAb [50,61]. Like this, the Fc:FcγR-mediated phagocytic uptake of PKH-26-labeled primary patient CLL target cells could be analyzed by detection of CD11b^+^PKH26^+^ cells via FACS analyses. This study emphasizes the contribution of ADCP during the treatment with α-CD20 therapeutic mAbs, since clinically applied pharmaceuticals blocking ADCP (inhibitors for BTK, Pi3Jδ or BCL2) were able to decrease ADCP in this model. Of note, also the contribution of complement to ADCP was indicated in this study, as ofatumumab, a mAb selected for increased complement activation, significantly increased ADCP in the presence of complement [50]. As an example, Rijkers et al. analyzed the FcγRII-mediated phagocytosis of PKH26-labeled platelets by monocyte-derived macrophages with and without inhibition of FcγRIIa by a blocking antibody or using an inhibitor for the downstream signaling molecule Syk. In this study, they unraveled the ability of HLA-specific autoantibodies to induce platelet-activation and in addition Fc-mediated depletion of platelets by macrophages and thereby contribute to the refractoriness to platelet transfusion [61].

#### 2.4.3. Other FcγR-Interaction/Binding Assays

As FcγR crosslinking assays, Jurkat cells expressing the respective receptor are incubated with the therapeutic mAb of interest (on ice) to be crosslinked by a specific F(ab’)2 fragment. At a given time point, cells are lysed and used for SDS-PAGE and Western blot (WB) with an α-phosphotyrosine-HRP antibody demonstrating the activation status of the FcγR-associated ITAM [54].

A further assay based upon crosslinking uses a competitive setting indicating homogenous proximity by luminescence. GST-tagged FcγRs are incubated with the mAb of interest (or controls). A mixture of glutathione acceptor beads (binding to GST-tag on the FcγR), biotinylated antibody (binding to the FcγR), and streptavidin-coated donor beads (binding on biotin on the antibody) will result in luminescence. Addition of the therapeutic mAb reduces the luminescence signal by outcompeting the biotinylated antibody of the system which makes the binding reflecting the potential for ADCP-induction quantifiable [30]. 

Another assay uses BW5147 mouse thymoma cells as reporter cells expressing one specific human FcγR, each. Upon binding to the Fc part of the according antibody, these cells secrete murine interleukin (IL)-2, which can be measured by ELISA [62,63]. In a recently published study, these reporter cells could be used to detect and quantify soluble but not immobilized immune complexes, which could provide a method to analyze and identify soluble immune complexes (sIC) in clinically relevant samples such as in SLE patients [64].

#### 2.4.4. FcRn Binding Assays

As mentioned in the introduction, the FcRn is not directly linked to the induction of effector function of therapeutic mAbs. However, it plays a crucial role in their PD/PK and mAb serum half-life. For this reason, several analyses exist determining the binding capacities of therapeutic mAbs to this receptor in order to characterize their expected life span in the body. Furthermore, mAbs specifically targeting FcRn themselves are under clinical development as approaches pursuing the reduction of pathological autoantibody half-lives [33,65]. 

To define the ability of therapeutic mAbs to interact with FcRn, competitive methods are commonly used. For example, 293 T cells expressing the FcRn can be incubated with a fluorescent Fc domain occupying the FcRn with a certain affinity. After addition of the therapeutic mAb of interest, the decrease of fluorescent cells is associated with the binding properties of the tested mAb [33]. Alternatively, mAb:FcRn interactions can also be analyzed via adapted crosslinking assays as described above [66].

A further receptor occupancy assay analyzing the binding of an FcRn-targeting antibody used human villous trophoblasts derived from primary placental material. Cells are incubated with the FcRn-blocking mAb M281. Subsequently, the percentage of remaining free FcRn is determined by detection of cells, which can be stained intra- and extracellularly with a VT645-labeled M281. The assay indicates saturation of the trophoblasts within 30–60 min. An additional perfusion model using primary placentas received directly after cesarean sections can reveal inhibition of the placental transfer adalimumab by co-transfused M281 in a time and concentration-dependent manner. This suggests that M281 could be a novel therapeutic for the treatment of fetal and neonatal diseases caused by transplacental transfer of alloimmune and autoimmune pathogenic IgG molecules [67].

## 3. Fc:FcγR Interaction-Mediated Side Effects

The FDA first approved mAb, Orthoclone OKT3 (muromonab), has been designed as murine IgG2a α-CD3 for immunosuppression in patients who received an organ transplant [68,69]. CD3 on T cells is internalized upon binding OKT3, thereby preventing inflammatory immune reactions. However, in several patients the mAb-induced severe and partially life-threatening side effects were caused by a cytokine release syndrome (CRS), which was induced by crosslinked CD3 on T cells via binding of FcγRs [70]. Due to the severe side effects, the manufacturer withdrew OKT3 from the market in 2010. Studies analyzing the effector functions revealed that a Leu235Glu mutation within the Fc part could prevent binding by FcγRs and is therefore considered to minimize the risk of CRS [70]. Additionally, an IgG4 variant of OKT3 showed a less inflammatory potential [70] highlighting the importance of taking Fc:FcγR interactions for therapeutic mAb design into account already back then. Accordingly, teplizumab shares the same binding site as OKT3 but is a humanized IgG1 mAb with amino acid changes at positions 234 and 235 to reduce Fc-mediated effector functions and is currently under clinical evaluation [71,72,73]. 

Probably the most prominent case of severe side effects has been observed during the first in-human clinical trial of TGN1412 in March 2006. TGN1412 is a humanized mAb of the IgG subclass 4 that specifically binds CD28 on (human) T cells. The mAb was initially designed for the treatment of RA or B-cell CLL by activating preferentially regulatory T cells [41,74]. During the clinical trial, shortly after application, the volunteers suffered from a life-threatening cytokine storm causing massive side effects that led to multi organ failure in all patients. Since then, several studies investigated this unforeseen phenomenon. Amongst other immune activating processes, also crosslinking of the TGN1412 Fc part has been observed to be critically involved in inducing cytokine secretion in vitro [41,75]. Even though TGN1412 was designed as IgG4 subclass to avoid excessive Fc:FcγR interactions, we were able to show that exactly these interactions induce strong T cell activation (T cell proliferation) in vitro [76]. It is generally well accepted and has been shown in several studies that increased Fc:FcγR interaction is related to increased Fc-mediated effector functions [25,77,78,79,80,81,82]. However, the results of our own studies indicate that the strength of the Fc:FcγR binding capacities do not necessarily correlate with the strength of the induced effector functions [76]. Thereby, especially FcγRIIa and FcγRIIb, known for weak binding capacities for IgG4 [83,84], which has been also confirmed in this context for TGN1412, were able to induce strong TGN1412-mediated T cell activation in vitro. However, FcγRI, with strong binding capacities for IgG4 including TGN1412, was not able to induce effector functions leading to T cell proliferation. In line with this, Ball et al. demonstrated enhanced effector function of an S228P-mutated TGN1412 variant (inhibiting Fab arm-exchange) without increased FcγR binding capacities [85]. 

Nevertheless, also for approved mAbs that are commonly used, side effects have been observed that are most likely mediated via unwanted interactions of the Fc part with FcγRs or complement components. For example, in rare cases, the α-CD20 mAb rituximab induces a CRS in some patients. In these cases, it has been observed so far that particularly patients with high tumor burden are affected. It is assumed that the excessive activation of the complement system and the subsequent lysis of the targeted CD20^+^ cells as well as the Fc:FcγR interactions with recruited macrophages lead to a strong cytokine secretion [86,87,88,89].

In general, it has been discussed that therapeutic mAbs or fusion proteins with an Fc part have the theoretical potential to induce a CRS via Fc binding to FcγRs on NK cells and neutrophils [25,90,91,92]. This potential might by increased when the mAb’s MoA is the induction of ADCC or CDC, especially when alterations for increased Fc binding are created [21]. Furthermore, the impact on a potential CRS might be also depending on the antigen-target of the mAb like, e.g., activating immune modulatory molecules on immune cells (such as CD3 or CD28 on T cells) or molecules with agonistic activity [25], which highlights the importance for a better understanding of Fc-mediated effector functions in vivo.

## 4. Novel Strategies for the Development of Therapeutic mAbs

In general, when it comes to the development of a therapeutic mAb, beside Fab specificity, the choice of the IgG subclass is always a major step dependent on the desired MoA. For therapeutic purposes, commonly IgG1, IgG2, and IgG4 mAbs are used. IgG1 is the most represented IgG subclass that is especially used for cancer treatment due to its ADCC- and CDC-mediating function [7]. The ability to mediate this effector function is even more pronounced for subclass IgG3 [10,84,93]. However, due to its shorter serum half-life, caused by an extended hinge region and lower binding affinity to the FcRn, IgG3 is currently unattractive for therapeutic purpose [84,94,95,96]. Subclasses IgG2 and IgG4 promote only minor cytotoxic effector functions and are consequently rather used when Fc-mediated effector functions are unwanted as MoA [83,94,95,97]. An example for this are checkpoint inhibitor mAbs for which ADCC and CDC activities are avoided. Approved anti-PD-1 mAbs such as camrelizumab, cemiplimab, nivolumab, or pembrolizumab are therefore chosen to be designed as IgG4 [98].

Beside the classical unmodified IgG subclasses, in the following referred to as wildtype (WT), IgA has recently been discussed as potential therapeutic mAb candidate [99]. IgA has been shown to have a more favorable gastrointestinal stability when compared to IgG [100]. Additionally, the high potential of IgA for neutrophil recruitment promoting killing of tumor cells, and the anti-viral activity mediated by neutralizing viruses such as rotavirus [101,102,103,104,105], poliovirus [106,107,108], influenza virus [109,110], or severe acute respiratory syndrome coronavirus 2 (SARS-CoV-2) [111], have been discussed [99]. However, the large majority of mAb engineering and Fc modifications have been investigated and discussed in several publications regarding IgG.

### 4.1. Engineering Methods to Address the FcγR Interaction

**Figure 4:**
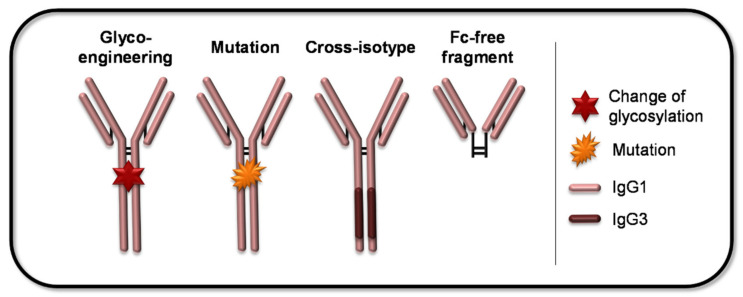
Possible mAb Fc-related modifications. The large variety of mAb-modifications concerning the Fc part can be grouped into four main categories, as illustrated with simplified representatives. The Fc-mediated effector functions can be influenced by changing the glycosylation-pattern of the mAb (glyco-engineering) or by introducing (point) mutations in the Fc domain. Furthermore, exchanges of distinct Fc domains with the respective domains derived from another IgG subclass are applicable to modify effector functions. The last category comprises the truncation of the Fc domain, which results in mAbs incapable of eliciting FcγR-mediated downstream events.

The above-mentioned examples in Section 3 made clear that novel strategies for the design of mAbs, especially with engineered Fc parts, are an advantage for a safer and more efficient therapy. Many studies investigated the role of Fc alterations in terms of the mediated effector function. Techniques regarding Fc-engineering can be often either used to enhance or to reduce the Abs Fc-mediated effector functions. For a better understanding, a simplified overview of possible Fc-related modifications is given in Figure 4.

Generally, Fc part binding to FcγRs or C1q of the complement system is engaged via the proximal CH2 domain and the lower hinge region [77,112,113,114,115,116,117]. To combine favorable properties of different IgG subclasses, cross-isotype antibodies have been generated. As mentioned before, IgG1 and especially IgG3 induce strong cytotoxic effector functions [49,84,95]. However, IgG3 is more efficient regarding effective complement activation via binding to C1q when compared to IgG1 [118]. For the creation of an Ab with even enhanced effector functions that bear both, high potential for ADCC and CDC, the CH2 as well as a part of the CH3 domain of an IgG1 have been switched with the respective domains of an IgG3 [119,120]. This cross-isotype antibody showed enhanced cytotoxic activities [119]. In contrast, for reduced effector functions, isotype switching has been investigated for IgG2 and IgG4 isotypes, which mediates only limited FcγR interactions [121]. Instead of replacing whole domains of the Fc part constant region, attempts have been undertaken to change only selected amino acids of different IgG subclasses [98,121]. One example that has been presented, is the mutant IgG2c4d (V234A/G237A/P238S/H268A/V309L/A330S/P331S), which was engineered by the exchange of residues of human IgG2 with human IgG4 residues. This modification could prevent binding of the mutant mAb by FcγR or C1q and therefore, the induction of ADCC, CDC, or ADCP when compared to the origin WT IgG2 [122,123]. In fact, investigated Fc-modification made it also from bench to clinic. The α-C5 mAb eculizumab is the first approved cross-isotype mAb, which has the amino acids 118–260 of IgG2 and the amino acids 261–447 of IgG4 resulting in limited or undetectable Fc-mediated effector functions [124].

The analysis of specific point mutations within IgG revealed further possibilities to adjust antibody effector functions. Investigated point mutation combinations such as Ser239Asp and Ile332Glu, or Gly236Ala, Ser239Asp, Ala330Leu, and Ile332Glu (described as GASDALIE) were shown to effectively increase FcγRIIIa binding affinities [125,126]. The same is true for the SDALIE mutation, which is a combination of mutations at Ser239Asp, Ala330Leu, and Ile332Glu [125,127,128]. Comparison during a clinical trial between the recently FDA-approved α-HER2 mAb margetuximab bearing combined mutations of Phe243Leu, Arg292Pro, Tyr300Leu, Val305Ile, and Pro396Leu (described as variant 18 [129]) and the α-HER2 mAb trastuzumab without mutations showed elevated clinical efficacy of margetuximab [130]. However, approved checkpoint inhibitors targeting PD-L1 such as durvalumab or atezolizumab, are designed as IgG1. Here, Fc parts include a triple mutation of L234F/L235E/P331S (for durvalumab) [131,132] preventing or limiting FcγR interactions or a mutation of N297A (for atezolizumab) which causes a loss of glycosylation limiting Fc-mediated effector functions [133,134].

It has been observed in several studies that the same mAbs which have only a difference within their glycosylation pattern bear different binding affinities for FcγRs [78,79,82,94,135,136,137]. The main structure of the glycan contains *N*-acetylglucosamine and mannose while the conserved gycosylation site is located at amino acid position N297 in the CH2 domain of the IgG’s Fc part [7,23]. Modifications of the Fc-related glycosylation pattern and the associated effects have been investigated very early to modify Fc properties such as mutations at N297 like N297A, N297Q, or N297G, which were shown to reduce Fc-mediated effector functions [138,139,140,141]. It has been discovered that glycosylation of the IgG’s Fc region critically affects Fc binding capacities by FcγR or complement. Alterations of the glycosylation pattern might therefore have a major impact on the safety and efficacy of a therapeutic mAb [82]. 

Changes in the glycosylation pattern are able to reduce or increase Fc-mediated effector functions, making this method an important tool for mAb therapy. Aglycosylation of mAbs leads to decreased cytotoxic effector functions and is used when ADCC or CDC is unwanted in cases where neutralizing and agonistic/antagonistic properties are chosen as MoA. However, it has been demonstrated by different studies that some substitutions within the CH2 or CH3 domain (or both) do not completely prevent Fc interaction of aglycosylated antibodies with some FcγRs [142,143,144,145,146]. 

In contrast to aglycosylated antibodies, it has been demonstrated that afucosylated antibodies, of which the core fucose has been removed, showed increased binding to FcγRIIIa resulting in increased ADCC activity [147,148,149,150,151]. It is suggested that the absence of fucose results in stronger interactions of the Fc part with the glycan of amino acid N162 in FcγRIIIa due to a reduced allosteric impact, thereby promoting the effector function [120,152]. The concept of afucosylation has been already implemented for therapeutic mAbs. The approved mAbs for cancer treatment mogamulizumab (α-CCR4) and obinutuzumab (α-CD20), as well as benralizumab (α-IL5R) for asthma treatment are non-fucosylated IgG antibodies with enhanced ADCC activity translating the advances of glyco-engineering into the clinic [153,154,155,156]. The combination of chemotherapy with e.g., obinutuzumab accomplished in patients with follicular lymphoma (FL) and CLL shows a higher efficacy when compared to supportive treatment with α-CD20 mAb rituximab [156,157,158]. However, it has to be taken into consideration that obinutuzumab, unlike rituximab, also induces apoptosis [159,160,161,162], which seems not to be Fc-meditated [159]. Thus, afucosylation of other already approved therapeutic mAbs could further increase ADCC properties as it has been shown for rituximab or trastuzumab [160,163].

Fc-mediated effector functions can also be affected by sialyation. FcγRIIIa binding affinities for sialylated Fc parts are clearly reduced [164,165,166]. Additionally, for mAbs, hypersialysation was shown to reduce binding properties, and consequently ADCC and CDC activity [164,165,167]. Thereby, it is suggested that the added sialic acid might affect the structure of the hinge region, which impacts binding to FcγRs [168,169]. Furthermore, it has been demonstrated by Bas et al. that hypersialylation of N297 is able to extend the half-life of IgG [170]. Glyco-engineering, regardless of use to enhance or reduce cytotoxic effector functions, is a promising tool not only for the design of future therapeutic mAbs but also to adjust already existing mAbs to increase effectiveness and reduce side effects.

### 4.2. Alternative Antibody Formats

Most of the (FDA-)approved mAb formats for therapy are full-size mAbs [96]. Even though in this full-size format the Fc part has the advantage of increasing the mAb stability, to allow multivalent binding, and to mediate effector functions as well as serum half-life [96], the downside is at the same time the large size and molecular weight (~150 kDa). Especially during treatment of solid tumors, it has been suggested that mAbs can only poorly penetrate the tumor tissue and that only 0.001–0.01% of the applied mAb would accumulate per gram of solid tumor [171,172]. This effect could be additionally promoted by a very high antibody:antigen affinity, limiting penetration of solid tumors and intratumoral diffusion [173]. The focus of further mAb engineering is therefore not only on alterations within the Fc part but also on new formats without an Fc part and a significantly altered structure. One class that has been brought up by the development of antibody engineering includes bispecific antibodies. This class contains various formats with and without Fc part. A developing field in cancer treatment to overcome the problem of inefficient tumor penetration is the creation of bispecific killer cell engagers (BiKEs) consisting of two linked variable portions, VH and VL (single chain variable fragment, scFv), respectively [174]. One of the scFv-fragments targets the tumor antigen and the other scFv-fragment targets FcγRIIIa on NK cells, thereby stimulating the NK cells not via classical engagement of FcγRIIIa but via α-FcγRIIIa binding. It is suggested that this specific FcγRIIIa binding without the influence of the FcγRIIIa allotype might lead to a stronger NK cell activation when compared to Fc:FcγRIIIa interaction [175,176]. Moore et al. were able to show an increased binding affinity and cytotoxicity for a bispecific α-HER2/α-FcγRIIIa antibody in comparison to a Fc:FcγRIIIa interaction of a conventional α-HER2 antibody [177]. Another advantage of direct α-FcγRIIIa binding is the avoidance of the competition between therapeutic mAbs and serum IgG, which might saturate the receptor [178,179,180]. BiKEs have been shown to successfully penetrate tumor tissue and to mediate cytotoxic effector functions by recruitment of immune effector cells [174,181,182,183]. As an example, for bispecific (b)scFv FcγRIII/CD19 an increased efficacy regarding NK cell-mediated effector function [174,184] has been observed.

Another strategy to engineer mAbs that avoids Fc-mediated effector functions is using antibody fragments lacking the whole Fc part. A Fab or F(ab’)2 fragment binds specifically to its target but cannot be engaged by FcγRs or complement. This concept is followed with the approved mAb Fab fragment certolizumab pegol (α-TNF-α) used for treatment of, e.g., Crohn’s disease or RA and is thought to reduce Fc-mediated side effects. Since Fab fragments loose stability due to lack of the Fc part and show therefore a significantly shorter half-life, which might be only a few hours or even minutes depending on their format [185,186], certolizumab pegol is conjugated to polyethylene glycol (PEGylated) which increases its half-life. 

Additionally, Fc-modifications can contribute to improved mAb’s serum half-life and are therefore another important aspect of enhancing mAb efficacy. In vivo binding of FcRn to the CH2-CH3 inter-domain region [187] is crucial to prolong half-life of mAbs which is for the most therapeutic mAbs > 20 days [188]. Analysis of the mutated Fc variant M252Y/S254T/T256E (YTE) as well as Fc variant M428L/N434S revealed that these variants were bound by FcRn with higher affinity in a pH-dependent manner leading to extended half-life when compared to WT IgG1(at pH 6.0) [189,190]. 

### 4.3. Further Strategies to Modify Fc:FcγR Interaction

Engineering and investigating therapeutic antibody variants with individually designed effector functions bearing a favorable safety and efficacy profile, requires many recourses in terms of time and costs [191,192]. Implementing new technologies and platforms will help in the future to further optimize this process. Recently, Chen et al. developed a mammalian cell display-based Fc-engineering platform [192]. A large number of Fc-variants was screened with mammalian cell display and next generation sequencing and thereby candidates with selectively enhanced affinities for FcγRIIIa or FcγRIIb were identified. According to the authors, the advantage of this platform is the high throughput of Fc parts that can be screened. Thus, this method can facilitate the identification of altered Fc parts in order to optimize Fc:FcγR interaction. Furthermore, newly identified Fc-variants can be substituted in nearly any antibody, which has been shown in the study for trastuzumab and rituximab, as well as for CD40 agonists. The selected antibodies with new identified Fc part showed a higher antitumor efficacy in in vitro and in vivo models in comparison to the original mAbs [192]. 

Besides all modifications that can be undertaken to alter Fc binding capacities and Ab function, it is important to note that additional factors have to be considered which are involved in vivo. It has been thereby discussed that also the antigen binding can have an influence on the Fc-mediated effector functions by affecting FcγR or C1q binding [193,194]. Complexed antigen:antibody engagement has been shown to critically increase Fc:FcγR interactions [195]. 

Even though antibodies are in the main focus regarding optimized Fc function, the FcγRs itself should also be taken into consideration. FcγRs also have certain glycosylation patterns that influence the Fc-mediated effector functions. These patterns might be different for the same FcγR when they are expressed on different cells [195]. For FcγRIIIa it has been shown that this receptor expressed by NK cells has more high-mannose and complex-type oligosaccharides when compared to a variant expressed by monocytes [196].

Furthermore, several studies showed that the different FcγR genotypes have a significant influence on Fc-mediated effects. FcγRIIIa, responsible for ADCC activity by NK cells, shows as polymorphic variant V158 higher binding affinities for IgG1 when compared to variant F158 [83,84,197,198]. In line with this, for the mAbs cetuximab, trastuzumab, and rituximab, a higher clinical efficacy has been observed in cancer patients with FcγRIIIa variant V158 than in patients with FcγRIIIa variant F158 [199,200,201]. For this reason, Barb et al. discussed the option of allogeneic NK cell transplants since also NK cells of a donor without a matching MHC are tolerated [202,203,204,205]. There are hints that allogeneic NK cells might have a reduced efficacy. A disintegrin and metalloprotease 17 (ADAM17)-mediated FcγRIIIa shedding, which normally occurs after FcγRIIIa binding to Fc, can be prevented via the FcγRIIIa variant S197P [206]. This prevention of receptor shedding may enable the receptor to bind new targets, however, it might also lead to a reduced NK cell survival and decreased cytotoxicity [207]. In other studies, blocking of ADAM17 in peripheral blood NK cells by CRISPR/Cas9 revealed enhanced IFN-γ levels and ADCC in the presence of rituximab-opsonized Raji target cells [208]. Additionally, the expression of non-cleavable FcγRIIIa in induced pluripotent stem cell derived human NK cells mediated higher cytokine levels and ADCC when compared to FcγRIIIa-WT expressing peripheral blood NK cells during culture with tumor cells and different therapeutic mAbs [209]. This concept is currently under clinical evaluation for the treatment of acute myeloid leukemia (AML) (NCT04023071) and patients hospitalized with hypoxia from coronavirus disease (COVID-19) (NCT04363346) [210]. In addition, other concepts such as human NK cell lines (NK92 cells) that express the FcγRIIIa polymorphic variant V158 and secret IL-2 (described as HaNK cells) [211] or NK cells expressing recombinant high-affinity FcγRI for enhanced ADCC activity [212] are currently being investigated to further increase efficacy of therapeutic mAbs. 

## 5. Conclusions and Further Directions

Even though FcR-mediated effector functions are evaluated in the context of efficacy of therapeutic mAbs with diverse approaches, some mechanisms of this complex context remain to be understood. With the gain of knowledge, safety and efficacy assessment of mAbs are continuously being optimized. Furthermore, in vivo milieu and different conditions during diseases need to be further investigated. The development of mAb design since the first approved mAb in 1986 has been already rapidly evolved and improved. This resulted in a large variety of therapeutic mAbs with different possible MoA. Further analyses will contribute to the design of safer therapeutic mAbs with fewer side effects and with an even higher efficacy profile in the future. The adjustment of the mAb’s effector function profile will also lead to an increase of possible applications. Although mAbs are a relatively “young” therapeutic product class, the accelerating gain of knowledge reveals the chance to develop and improve treatment strategies for a broad range of immunological conditions.

## Figures and Tables

**Table 1:** Overview of FcγRs.

	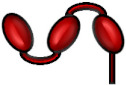	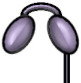	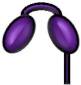	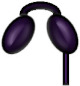	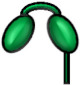	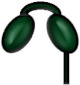
Name	FcγRI	FcγRIIa	FcγRIIb	FcγRIIc	FcγRIIIa	FcγRIIIb
**CD**	CD64	CD32a	CD32b	CD32c	CD16a	CD16b
**Gene**	*FCGR1A*	*FCGR2A*	*FCGR2B*	*FCGR2B*	*FCGR3A*	*FCGR3B*
**Affinity**	High	Low to medium	Low to medium	Low to medium	Low to medium	Low to medium
**Major role**	Activation	Activation	Inhibition	Activation	Activation	Decoy Activation
**Human IgG interaction**	IgG1, IgG3, IgG4	IgG3, IgG1, IgG2, IgG4	IgG3, IgG1, IgG4	IgG3, IgG1	IgG1, IgG3, IgG4	IgG1, IgG3
**Cell type**	Monocytes DCs (Neutrophils) (Mast cells) (Macrophages)	Monocytes Neutrophils DCs Macrophages Basophils Eosinophils Mast cells Platelets	B cells Monocytes DCs Macrophages Eosinophils Basophiles (Neutrophils) (Activated T cells)	NK cells Monocytes Neutrophils	NK cells Monocytes (Macrophages) (Activated T cells)	Neutrophils (Basophils)
